# Treatment planning evaluation and experimental validation of the magnetic resonance-based intrafraction drift correction

**DOI:** 10.1016/j.phro.2024.100580

**Published:** 2024-04-21

**Authors:** Madelon van den Dobbelsteen, Sara L. Hackett, Bram van Asselen, Stijn Oolbekkink, Bas W. Raaymakers, Johannes C.J. de Boer

**Affiliations:** Department of Radiotherapy, University Medical Center Utrecht, Heidelberglaan 100, 3584 CX Utrecht, The Netherlands

**Keywords:** MR-guided radiotherapy, Motion management, Intrafraction drift correction, Intrafraction motion

## Abstract

**Background and purpose:**

MRI-guided online adaptive treatments can account for interfractional variations, however intrafraction motion reduces treatment accuracy. Intrafraction plan adaptation methods, such as the Intrafraction Drift Correction (IDC) or sub-fractionation, are needed. IDC uses real-time automatic monitoring of the tumor position to initiate plan adaptations by repositioning segments. IDC is a fast adaptation method that occurs only when necessary and this method could enable margin reduction. This research provides a treatment planning evaluation and experimental validation of the IDC.

**Materials and methods:**

An in silico treatment planning evaluation was performed for 13 prostate patients mid-treatment without and with intrafraction plan adaptation (IDC and sub-fractionation). The adaptation methods were evaluated using dose volume histogram (DVH) metrics. To experimentally verify IDC a treatment was mimicked whereby a motion phantom containing an EBT3 film moved mid-treatment, followed by repositioning of segments. In addition, the delivered treatment was irradiated on a diode array phantom for plan quality assurance purposes.

**Results:**

The planning study showed benefits for using intrafraction adaptation methods relative to no adaptation, where the IDC and sub-fractionation showed consistently improved target coverage with median target coverages of 100.0%. The experimental results verified the IDC with high minimum gamma passing rates of 99.1% and small mean dose deviations of maximum 0.3%.

**Conclusion:**

The straightforward and fast IDC technique showed DVH metrics consistent with the sub-fractionation method using segment weight re-optimization for prostate patients. The dosimetric and geometric accuracy was shown for a full IDC workflow using film and diode array dosimetry.

## Highlights

• Magnetic resonance guided intrafraction drift correction was researched.
• Target coverage benefits of intrafraction adaptation methods were shown.
• Two adaptation methods both showed a median target coverage of 100.0% for 55 fractions.
• The experimental results verified the new method with a gamma passing rate of 99.1%.

## 1 Introduction

Magnetic Resonance (MR)-guided online adaptive treatments enable better targeting of the tumor and give the opportunity to account for interfractional variations, which could potentially reduce toxicity in high-risk prostate patients [1], [2], [3], [4], [5]. Intra-fraction motion up to 1 cm in prostate patients was demonstrated using real-time imaging [6], [7], [8], [9], [10]. This intrafraction motion deteriorates treatment accuracy, especially when treatment times become longer when applying hypo-fractionation [11], [12]. Consequently, there is a need for adaptation methods to account for intrafractional changes to reduce risk of toxicity [9].

Recently, an intrafraction adaptation method, sub-fractionation, was introduced for prostate Stereotactic Body Radiation Therapy (SBRT) patients [13]. The daily dose is delivered in two sequential parts (sub-fractions), each adapted to the latest anatomy. Imaging and treatment planning are executed in parallel, enabling an efficient and effective workflow. Due to reduced intrafraction translational motion, margin reduction was feasible, leading to a Clinical Target Volume (CTV) to Planning Target Volume (PTV) margin reduction from 5 mm isotropic margins to implemented anisotropic margins of 2 mm (Left–Right (LR) and Superior-Inferior (SI)) and 3 mm (Anterior-Posterior(AP)) [13]. The sub-fractionation occurs exactly mid-treatment for each patient. However, for some patients no intrafraction adaptation is needed, for others multiple adaptations per fraction are desired. Approximately 10% of the patients treated with sub-fractionation showed large intrafraction motion, where the reduced margins were insufficient. Preferably, the intrafraction adaptation occurs only when necessary, instead of one adaptation mid-treatment for each patient, leading to a more efficient workflow. Therefore, there is a need for patient-specific intrafraction adaptation methods guided by real-time imaging.

A new method was recently introduced to continuously monitor the tumor position during treatment delivery [14], [15]. The treatment plan can be adapted in one minute by repositioning the segments without optimization steps [16], denoted an Intrafraction Drift Correction (IDC). For upper abdomen patients an improved congruence to planned dose was shown using gating and IDC [17]. IDC is a valuable functionality for fast and patient-specific intrafraction adaptations, which could enable reduction of margins for prostate patients (or other treatment sites) as shown earlier with sub-fractionation.

Even though IDC is a valuable functionality there was a need for a treament plannning and experimental validation of this novel method. Therefore, an in silico treatment planning evaluation without intrafraction adaptation and with intrafraction adaptations, consisting of IDC and sub-fractionation using segment weight re-optimization as a benchmark, was simulated for 13 prostate SBRT patients for MR-based adaptive treatments. Additionally, the IDC was experimentally verified comparing measurements and calculations using film and diode array dosimetry. The goal of this research was to show a treatment planning evaluation and experimental validation of the IDC.

## 2 Materials and methods

### 2.1 Adaptation workflow and infrastructure

**Fig. 1:**
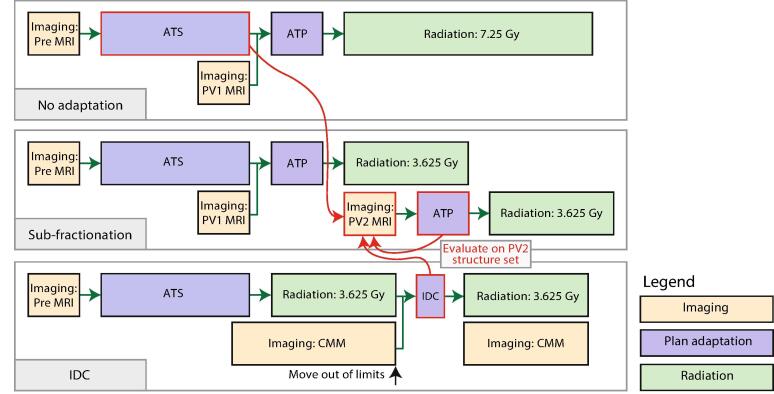
Workflow without intrafraction plan adaptation and with intrafraction plan adaptation, consisting of the sub-fractionation and Intrafraction Drift Correction (IDC) mid-treatment. The red colored adaptation steps between the methods are compared on the contours of the PV2 scan. Abbreviations: Adapt-To-Shape (ATS), Adapt-To-Position (ATP), Intrafraction Drift Correction (IDC), Pre-treatment (Pre) and Position Verification (PV).

During an interfraction adaptation scheme for prostate patients at University Medical Center (UMC) Utrecht treated on the 1.5 T MR linear accelerator (MR-linac), a T2-weighted offline MR-scan and reference plan were made. The contours were propagated from the T2-weighted reference MR-scan to the daily online Pre-treatment (Pre) scan. The contours were manually adapted where necessary. A treatment plan was optimized from fluence based on the daily adapted contours [2]. An extra Position Verification (PV1) MR-scan was acquired. An automatic match based on the tumor region was performed of the Pre and PV1 scans, and modified if necessary. If the displacement was larger than 1 mm, the pre-treatment plan was adapted with the Adapt-To-Position (ATP) method with the ’optimize weights from segments’ option, effectively shifting the dose distribution [2]. This workflow without intrafraction adaptation during beam-on is illustrated in Fig. 1.

Since December 2021 the intrafraction sub-fractionation method was used in UMC Utrecht, dividing each fraction of 7.5 Gy into two sub-fractions of 3.625 Gy, for prostate SBRT treatments as described by Willigenburg et al. [13]. During radiation of the first sub-fraction, Position Verification (PV2) imaging and treatment planning (ATP) including segment weight re-optimization was executed, enabling an efficient workflow with a treatment scheme of 10x3.625 Gy, illustrated in Fig. 1.

Another technique, comprehensive Motion Monitoring (CMM) (Elekta AB, Stockholm, Sweden) enables continuous monitoring of the tumor position during treatment delivery. CMM imaging involves 2D balanced cine MR-scans (6 Hz, interleaved coronal/sagittal planes) [14], [15]. The first sixty frames of the cine MR-scans are used for initialization of the 2D-3D registration template. The initial template registration to the corresponding planes of the 3D pre-scan is reviewed and if necessary adjusted. Next, all incoming cine MR images are registered to this template. When the tumor location shifts above a pre-defined limit, the plan can be adapted by repositioning the segments not yet delivered, denoted by IDC. This method uses Segment Aperture Morphing (SAM) to move the segments but does not include optimization. The created IDC treatment plan was checked and approved by the user in Monaco and the total workflow took only one minute from start of the procedure to plan delivery.

This in silico study simulated the workflow without intrafraction plan adaptation and the IDC for patients who had been treated with the sub-fractionation workflow. IDC modifications were simulated for patient shifts occurring exactly mid-treatment, consequently, CMM was not used. In the CMM simulation, illustrated in Fig. 1, an IDC was simulated mid-treatment regardless of the shift observed, corresponding to a 0 mm limit.

### 2.2 Patient cohort

Patient data were collected retrospectively under the FAST-ART protocol (IRB reference: 20–519/C). The patient cohort consisted of 13 patients and was selected to include patients with extreme translations and large intrafraction motion. The translations between PV1 and PV2 MR-scans were (partly) documented for prostate SBRT patients who were treated between May and December 2022. To include the extreme translations, patients were selected with at least one of the five fractions with minimum or maximum translations in LR, SI, AP or 3D directions, resulting in five patients. Eight extra patients were added to cover the full range of translations. The 3D translation ranged from 0 to 7.8 mm between PV1 and PV2 scans and from 1.2 to 15.8 mm between Pre and PV2 scans, illustrated in Supplementary Material A. Some fractions showed negligible translation between Pre and PV2 scans, requiring no plan adaptation for the sub-fractionation method. These fractions were excluded from the analysis, leading to 55 fractions over 13 patients. For most fractions, margins of 2 mm (LR and CC) and 3 mm (AP) between CTV and PTV were used. For 14 out of 55 fractions a larger isotropic margin of 5 mm was used due to larger shifts in earlier fractions of that patient.

### 2.3 Treatment planning

**Fig. 2:**
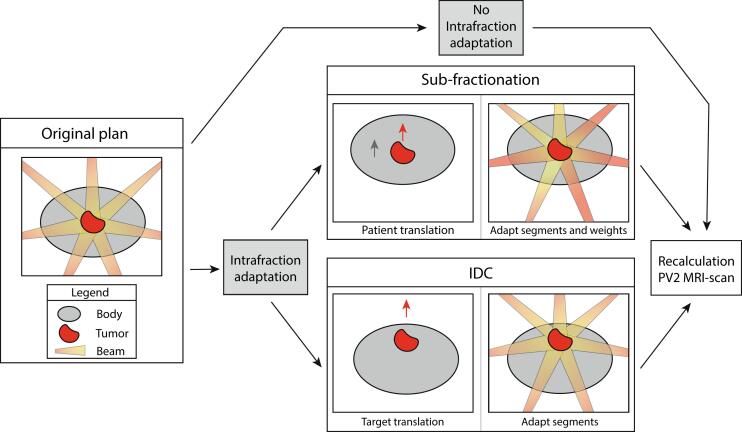
Schematic illustration of the treatment planning without intrafraction plan adaptation and with intrafraction plan adaptation consisting of the sub-fractionation and the Intrafraction Drift Correction (IDC).

An in-house template for prostate patients with seven IMRT beams was used in Monaco (v5.51.10). As part of the sub-fractionation workflow, an ATP step was used, whereby the segments were modified based on translations between Pre and PV2 MR-scans. Afterwards the weights were modified to optimize the dose shift to this new target position. The sub-fractionation treatment plan was calculated based on the Pre scan anatomy. To simulate the IDC process, ATP was used, only using adapt segments, and no further segment weight optimization. SAM was used to account for the new projection of the target position, but did not account for change of radiological target depth, SSD or off-axis position. The plan used for IDC analysis was based on a new, synthetic anatomy, where only the tumor was shifted according to the shift between Pre and PV2 scans, illustrated in Fig. 2. To compare DVH metrics without and with intrafraction plan adaptations on the same anatomy, the treatment plans were recalculated on the PV2 MR-scan. Contours were deformably propagated from Pre to PV2 scans, and within a margin of 2.5 cm in LR and AP directions, and 1.5 cm in SI direction outside the CTV contours were manually adapted where necessary. The treatment plans were calculated on a dose grid of 3 mm and a statistical uncertainty of 3% per control point.

### 2.4 DVH metrics

For a comparison between the adaptation methods, DVH metrics were used in Monaco with an interpolated grid of 1 mm. The clinical goals and acceptable limits of the sub-fractionation template are shown in Table 1. During plan optimization the aim was to reach the clinical goals, however with the acceptable limits the plan was still accepted without an extra check of the physician. Differences between sub-fractionation and IDC treatment plans were tested for significance using a two-tailed Wilcoxon signed-ranked test. Rejection of the null hypothesis (no difference between IDC and sub-fractionation DVH metrics) was set to p<0.01 due to the Bonferroni correction [18].

**Table 1:** DVH metrics for the treatment planning evaluation. V_95%_ represents 95% of the prescription dose (3.625 Gy).

Structure	Clinical goals	Acceptable limits
CTV	V_95%_> 99.0%	V_95%_> 98.9%
PTV	V_95%_> 99.0%	V_95%_> 96.0%
Rectum	$D_{1{\mathrm{cm}}^{3}}$< 38.0 Gy	
Bladder	$D_{5{\mathrm{cm}}^{3}}$< 37.0 Gy	
Sphincter	$D_{\mathrm{mean}}$< 20.0 Gy	

### 2.5 Film dosimetry

**Fig. 3:**
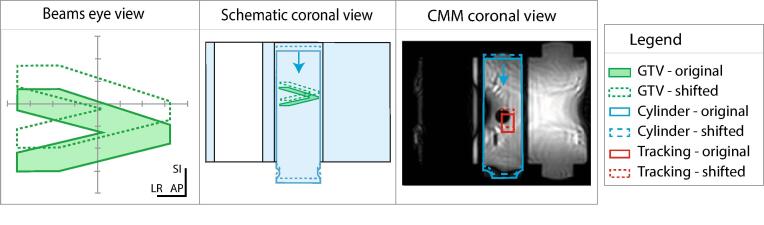
Overview experimental verification of the Intrafraction Drift Correction (IDC). The beams eye view including Gross Target Volume (GTV), schematic coronal view and Comprehensive Motion Monitoring (CMM) in the coronal view are illustrated using original and shifted structures.

To experimentally validate the IDC procedure, its dosimetric and geometric accuracy was verified using the QUASAR MRI^4*D*^ motion phantom (IBA QUASAR, Modus Medical Devices Inc., London ON, Canada), denoted as QUASAR phantom, containing EBT3 film (Ashland, Inc., Wilmington, United States). A treatment was mimicked using an IDC mid-treatment, illustrated in Fig. 3. A treatment of 14 beams, based on a rescaled lung SBRT template, was used in Monaco (v6.2.0.0) with a prescription dose of 7.5 Gy to match the dosimetric range of EBT3 film. The QUASAR phantom comprised an external body and two internal cylinders. The central cylinder contained EBT3 film (LOT 01042103) and was attached to a motor for movement during the treatment. The asymmetrical Gross Tumor Volume (GTV) with high dose gradients along the SI axis allowed clear registration of measured and calculated dose distributions and was useful for identifying possible errors in the displacement of the IDC plan. CMM software uses a tracking structure to identify displacements during the treatment. A rectangle around a part of an MR-visible sphere was used as the tracking structure, illustrated in Fig. 3. To mimic an IDC, the cylinder was moved 1 cm inferiorly mid-treatment, between beam 7 and 8. CMM software identified the displacement of the tracking structure and since the defined tolerance was exceeded, delivery was automatically paused. The segments were moved according to the shift, and the final seven beams were delivered with the IDC treatment plan. Treatment plans were recalculated on a fine dose grid of 1 mm with a statistical uncertainty of 0.5% per control point. The total calculated dose was created by summing the original plan dose distributions for the first seven beams and the IDC plan dose distribution shifted 1 cm superiorly for the final seven beams. Measured and calculated dose was compared, investigating dose differences, global γ values (2%/2 mm) [19] calculated with the CalcGamma function [20], and dose profiles.

### 2.6 Diode array dosimetry

The treatment delivered to the QUASAR phantom was irradiated on the Delta4 Phantom + MR (ScandiDos AB, Uppsala, Sweden), denoted as Delta4 phantom, for plan quality assurance (QA) purposes [21]. The Delta4 phantom remained in the original position for the first seven beams and the phantom was shifted 1 cm inferiorly for the final seven beams. As default, the treatment plans were calculated on a dose grid of 3 mm and a statistical uncertainty of 3% per control point for the original plan and a dose grid of 3 mm and a statistical uncertainty of 2% per calculation for the IDC plan. The total calculated dose was created by summing the original plan dose distributions for the first seven beams and the interpolated IDC plan dose distribution shifted 1 cm superiorly for the final seven beams. Measured and calculated dose was compared using global γ passing rates (3%/3 mm).

## 3 Results

### 3.1 In-silico treatment planning evaluation

**Fig. 4:**
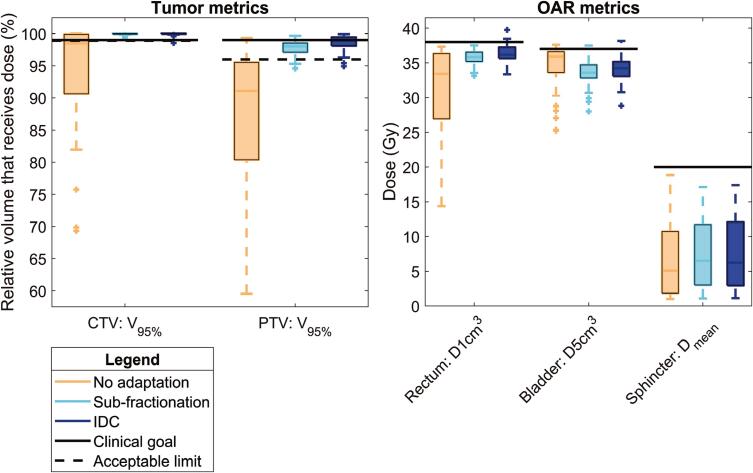
DVH metrics without intrafraction plan adaptation and with intrafraction plan adaptation consisting of sub-fractionation and the Intrafraction Drift Correction (IDC). Margin reductions were implemented for the PTV structure, only for 14 out of 55 fractions a larger isotropic margin of 5 mm was used due to larger shifts in earlier fractions of that patient. It should be noted that if no intrafraction adaptation was available, 5 mm isotropic margins rather than reduced margins would have been used.

The median CTV coverage [and ranges] without intrafraction adaptation (98.5% [69.3% 100%]) was much lower compared to CTV coverage of sub-fractionation (100.0% [99.3% 100.0%]) and IDC (100.0% [98.6% 100.0%]), illustrated in Fig. 4. It should be noted that if no intrafraction adaptation was available, 5 mm isotropic margins rather than reduced margins would have been used. The IDC technique showed DVH metrics consistent with the sub-fractionation method mid-treatment. In general, the IDC showed a significantly better coverage of the PTV, however higher doses were shown for rectum and bladder. The median [and ranges] percentage differences between IDC and sub-fractionation were 0.0% [-1.2% 0.4%], 0.7% [-2.0% 3.3%], 1.5% [-2.6% 5.9%], 1.8% [-2.3% 7.9%] and 1.5% [-9.0% 8.8%], for CTV, PTV, rectum, bladder and sphincter, respectively. Significant differences were found for the PTV (p<0.01), rectum (p<0.01) and bladder metrics (p<0.01). No significant differences were found for CTV (p = 0.06) and sphincter metrics (p = 0.03). The sub-fractionation method failed the acceptable limits for four and one fraction(s) out of 55 fractions for the PTV and bladder metrics, respectively. The IDC method failed the acceptable limits for one, two, four and three fraction(s), for the CTV, PTV, rectum and bladder metrics, respectively. Nevertheless, the dose differences were clinically irrelevant, as the IDC showed a slightly higher PTV coverage but also higher organs at risk (OARs) doses and the few fractions that do not meet the acceptable limits were only slightly outside the tolerances. No trend was shown between 3D translations and larger dose differences between IDC and sub-fractionation outcomes, illustrated in Supplementary Material B.

### 3.2 Experimental verification IDC

**Fig. 5:**
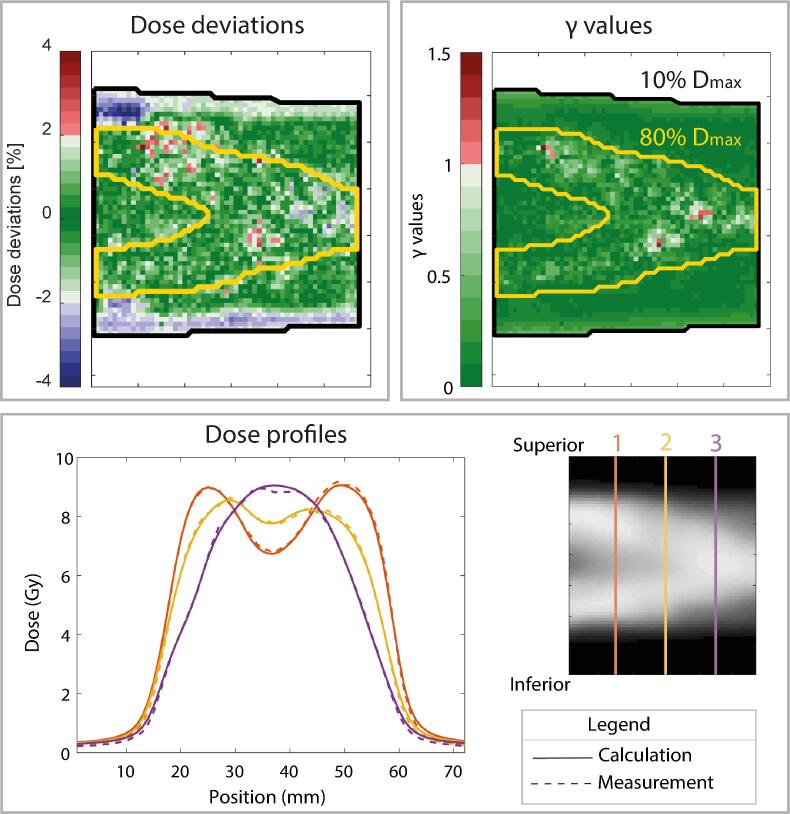
Dose deviations, γ values and dose profiles for the experimental verification of the intrafraction drift correction.

Experimental verification of the IDC showed good agreement between measurements and calculations based on the dose deviation, γ values and differences in dose profiles, illustrated in Fig. 5. The mean (± standard deviation) dose differences were −0.3% ± 1.2% and 0.1% ± 1.1%, for threshold doses of 10% and 80% of the maximum dose. γ passing rates were 99.6% and 99.1%, for the same threshold doses. On the Delta4 phantom the total delivered dose of the IDC workflow showed a γ passing rate of 99.6%.

## 4 Discussion

The in silico planning study showed the benefits of intrafraction adaptation methods, where the IDC technique showed DVH metrics consistent with the sub-fractionation mid-treatment. The experimental results verified the IDC methods, showing a good agreement between the measured and calculated IDC dose with high γ passing rates and small dose deviations.

For the in silico evaluation, the situation was mimicked where the treatment was adapted mid-treatment. Our in silico planning study showed the benefits of intrafraction adaptation methods. Grimbergen et al. also reported the dosimetric benefits for the first patients treated with gating and IDC [17]. They showed a better congruence to the planned dose regarding target coverage and OARs sparing. The sub-fractionation method takes approximately five minutes, therefore only one adaptation is feasible while maintaining an efficient workflow. However, for some patients multiple adaptations might be desired, which is feasible with the IDC. The selected patient group covered the full range of translations, results indicated that the full range of translations can be adequately addressed with IDC. The IDC showed a better coverage of the PTV and higher doses for the rectum and bladder compared to the sub-fractionation method. This finding contradicts research illustrating lower coverage of the PTV for adapt segments compared to adapt segments and weights [22]. In our research no weight optimization was used for the IDC and weights were optimized for the sub-fractionation adaptation method. Resulting in slightly higher OAR doses and a higher PTV coverage for IDC. However, as the DVH metrics are consistent for IDC and sub-fractionation for an adaptation mid-treatment, the use of IDC could also enable margin reduction for Unity treatments, as shown with sub-fractionation [13]. The option to perform multiple intrafraction adaptations could allow for yet further reduction of margins. However, a new anatomy was created for the IDC plan with a shifted tumor corresponding to the shift between Pre and PV2 scans. These shifts were deduced from the registration between 3D scans. In the realistic IDC scenario, the shifts are determined according to the 2D cine MR-scans including 2D-3D conversions. Both the higher spatial uncertainty of the 2D cine MR-scans and the 2D-3D conversions, could lead to higher uncertainties in the determined tumor shift [14], [15].

A good agreement between the total measured and calculated IDC treatment was shown with film and diode array dosimetry. Similar γ passing rates for relative dosimetry, ranging from 94.5% to 99.9% were shown for single fraction ATS procedures [23], compared to a γ passing rate of an IDC adaptation of 99.6% for absolute dosimetry without rescaling for threshold doses of 10% of the maximum dose. This indicates accurate outcomes of the experimental verification of the IDC procedure. Uijtewaal et al. measured the dose of a simulated treatment with prostate motion with and without IDCs using both film dosimetry and multiple plastic scintillation detectors [24]. Applying IDCs showed a better congruence with the planned dose compared to no adaptations with maximum dose differences of -5% and -18%, respectively. In our research, the total dose was determined by calculating the total planned dose including adaptations. Treatment plans of the diode array measurements could be improved by decreasing the grid size and statistical uncertainty.

One of the biggest advantages of the IDC is the patient-specific approach. Since the adaptation method takes less than one minute it is feasible to perform multiple adaptations per fraction. Especially with hypo-fractionation schemes a longer delivery time could lead to more intra-fraction motion and consequently the need for more adaptations per fraction. However, hypo-fractionation leads to a lower cost burden for the health care system and increased patient convenience [25], [26]. The Hermes trial tests the two-fraction treatment capabilities of the MR-linac for prostate patients [12], [27]. Our research focused on IDC and sub-fractionation, however other intrafraction adaptation techniques or interruptions, such as respiratory and exception gating could be used [17], [28]. The intrafraction adaptation methods should be verified experimentally using dedicated QA methods in real-time. Ongoing research shows the feasibility of deformable image registration and dose accumulation in real-time using cine MR-scans and linac logfiles, including determination of the accumulation error [9], [10], [29], [30], [31], [32].

In conclusion, the in silico planning study showed the target coverage benefits of intrafraction adaptation methods, where the straightforward and fast IDC technique showed DVH metrics consistent with the sub-fractionation method using segment weight re-optimization mid-treatment. The dosimetric and geometric accuracy was shown for a full workflow of the IDC using film and diode array dosimetry.

## CRediT authorship contribution statement

**Madelon van den Dobbelsteen:** Conceptualization, Methodology, Software, Validation, Formal analysis, Investigation, Data curation, Writing – original draft, Writing – review & editing, Visualization. **Sara L. Hackett:** Conceptualization, Methodology, Validation, Writing – review & editing, Supervision. **Bram van Asselen:** Conceptualization, Methodology, Validation, Writing – review & editing, Supervision. **Stijn Oolbekkink:** Investigation, Writing – review & editing. **Bas W. Raaymakers:** Conceptualization, Methodology, Validation, Writing – review & editing, Supervision, Funding acquisition. **Johannes C.J. de Boer:** Conceptualization, Methodology, Validation, Writing – review & editing, Supervision.

## Declaration of competing interest

The authors declare the following financial interests/personal relationships which may be considered as potential competing interests: The authors acknowledge funding by the Dutch Research Council (NWO) through Project No. 18495 (ADEQUATE).
