# Supplementary material for: Treatment planning evaluation and experimental validation of the magnetic resonance-based intrafraction drift correction
Source: Phys Imaging Radiat Oncol. 2024 Apr 21;30:100580. doi: 10.1016/j.phro.2024.100580 (PMC11068926; doi:10.1016/j.phro.2024.100580)
Supplement: Supplementary data 1 [file mmc1.pdf]

Supplementary Material: Treatment planning evaluation and experimental validation of the magnetic resonance-based intrafraction drift correction

Madelon van den Dobbelsteen, Sara L. Hackett, Bram van Asselen, Stijn Oolbekkink, Bas W. Raaymakers, Johannes C.J. de Boer

A. 3D translations

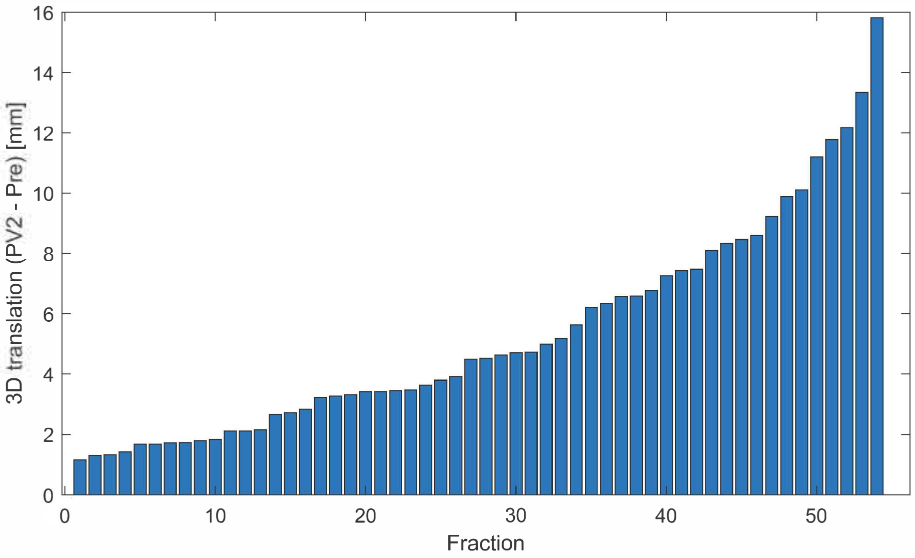

Figure A.1: 3D translations (PV2 - Pre) for all fractions. The patient cohort consisted of 13 patients and 55 fractions, and was selected to include patients with extreme translations and large intrafraction motion. The 3D translation ranged from from 1.2 to 15.8 mm between Pre and PV2 scans.

B. 3D translations versus DVH metrics

The DVH metrics for the Intra-fraction Drift Correction (IDC) and the sub-fractionation were plotted against the 3D shifts (PV2 - Pre). The 3D shifts were rounded to an integer, to group the dosimetric outcomes. The lines were connected between the median values of the boxplots of each 3D shift.

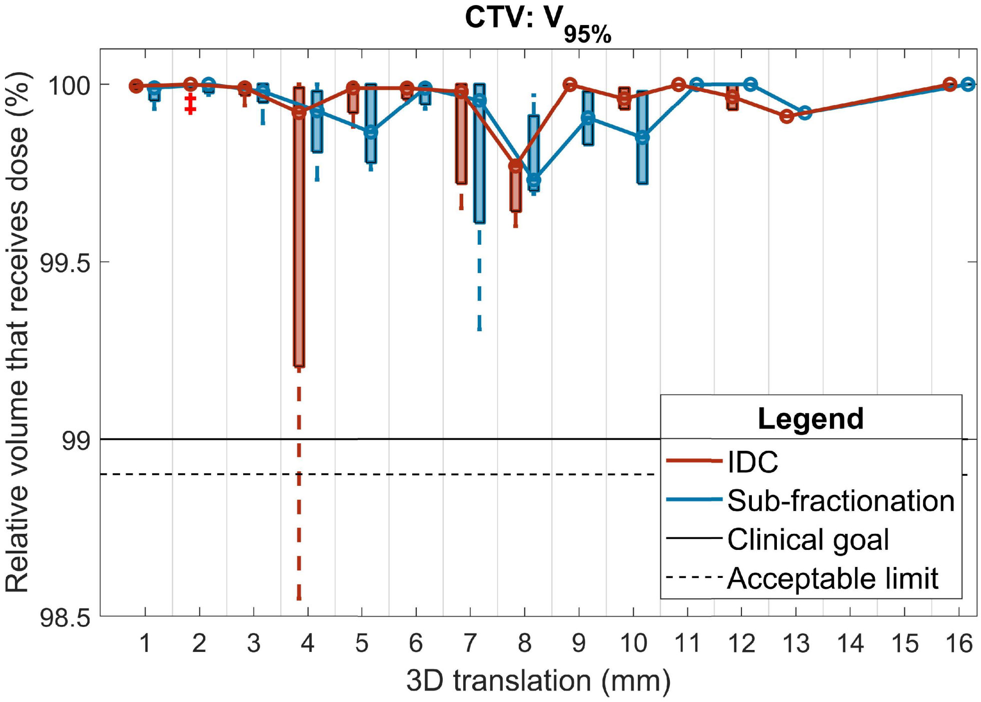

Figure B.1: 3D translations versus relative volume that receives dose for the CTV.

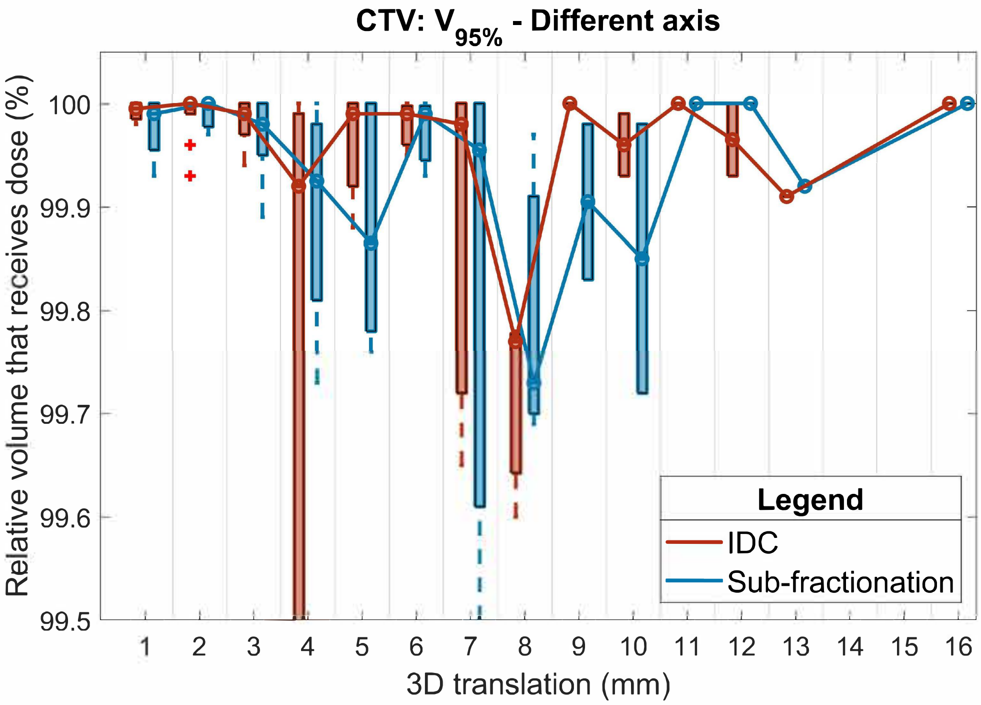

Figure B.2: 3D translations versus relative volume that receives dose for the CTV zoomed in y axis.

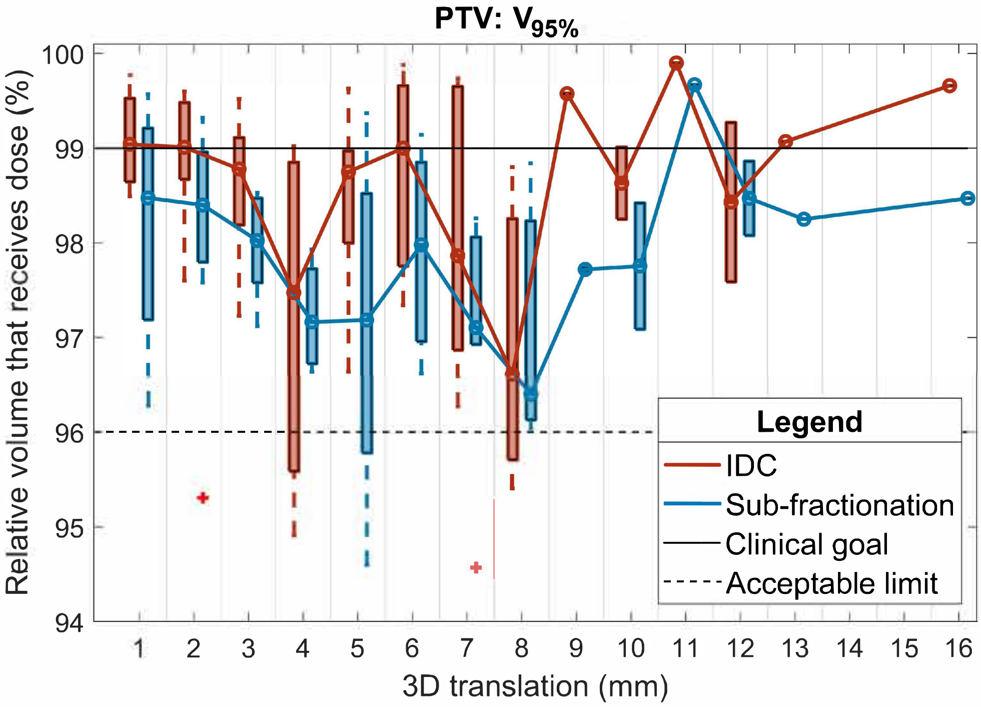

Figure B.3: 3D translations versus relative volume that receives dose for the PTV.

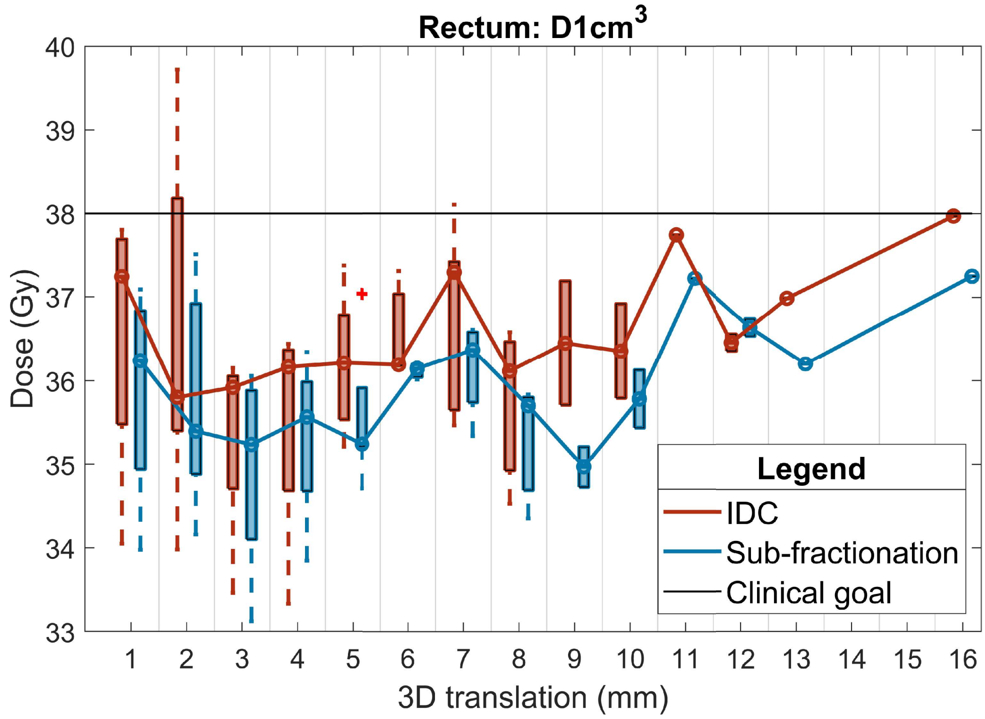

Figure B.4: 3D translations versus dose for the rectum.

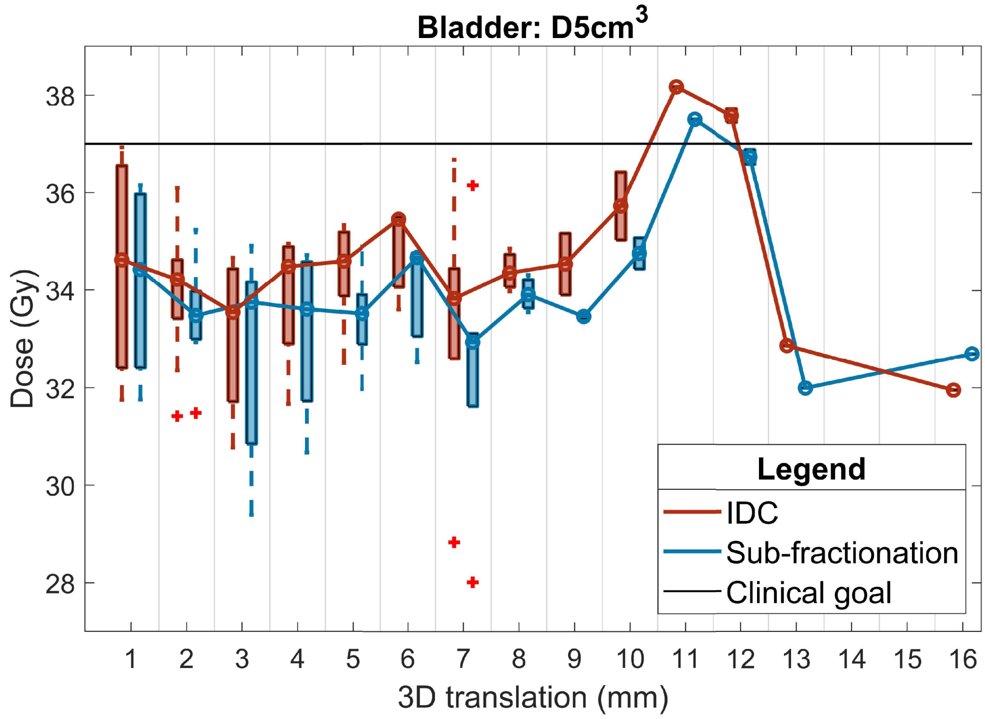

Figure B.5: 3D translations versus dose for the bladder.

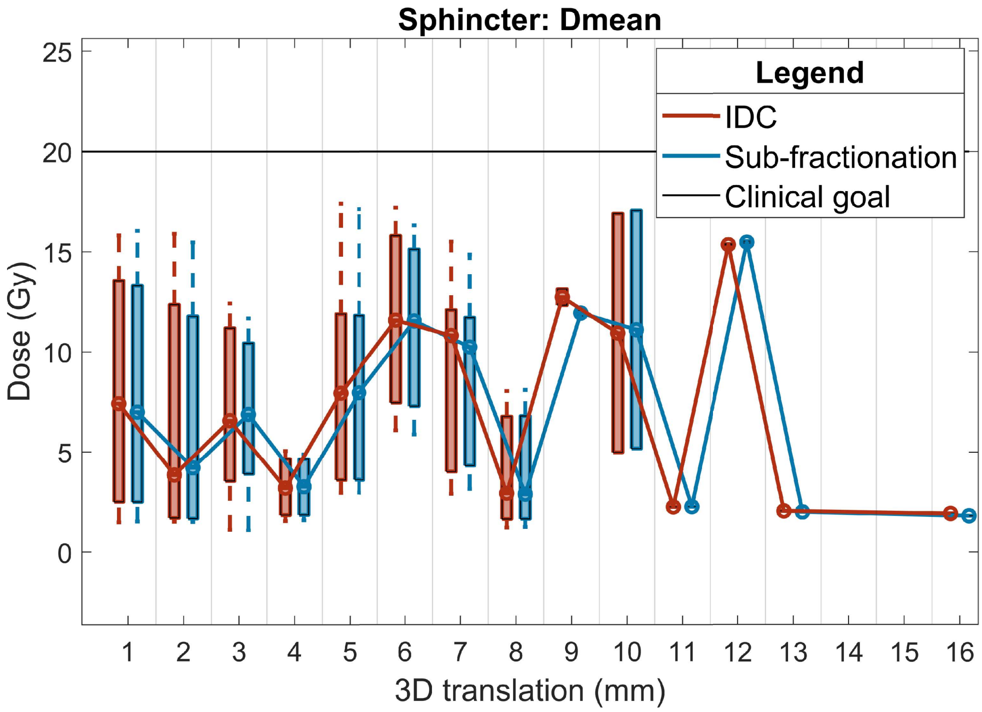

Figure B.6: 3D translations versus dose for the sphincter.
